# Different Shades—Different Effects? Consequences of Different Types of Destructive Leadership

**DOI:** 10.3389/fpsyg.2018.01289

**Published:** 2018-07-25

**Authors:** Ellen A. Schmid, Armin Pircher Verdorfer, Claudia V. Peus

**Affiliations:** TUM School of Management, Technische Universität München, Munich, Germany

**Keywords:** destructive leadership, differential effects, dark side of leadership, exploitative leadership, turnover intention

## Abstract

Destructive leadership comes in many shapes and forms. From reviewing the literature, we conclude that three major forms of destructive leader behaviors are described: (1) follower-directed destructive behaviors, i.e., genuine abusive forms of destructive leadership, (2) organization-directed behaviors, i.e., behaviors such as stealing from the organization or embezzlement, and (3) self-interested destructive leader behavior, i.e., leader who exploit others to reach their goals. One can easily imagine that these three types of leader behavior have very different effects on followers. Unfortunately, so far, there is no empirical evidence to support this, since comparative research in the field of destructive leadership is scarce. With this paper, we aim to address this gap: In two studies, an experimental and a field study, we examine the differential impact of these three different destructive leader behaviors on two important outcomes: first, their impact on different emotional reactions of followers, the most proximal outcome to a social interaction. Second, we examine a key outcome in leadership research: followers' turnover intention. The results suggest that different types of destructive leader behavior do impact followers differently. Whereas all three behaviors had a positive relationship with negative affect, follower-directed destructive behaviors had the strongest relation out of the three. As expected, all three types of destructive behavior relate to turnover intention, yet, the results of our study suggest that different types of destructive leader behavior relate to different urgencies of turnover intention. We conclude that a tailored approach to destructive leadership, whether in research or practice, seems necessary, as diverse types of destructive leader behaviors affect employees differentially.

## Introduction

The media frequently reports stories about so-called “bad bosses.” On a closer look, these destructive leader behaviors come in many forms. Recently, Jeff Bezos, CEO of Amazon, was announced as winner of the world's “worst boss” award at the 3rd International Trade Union Confederation World Congress in Berlin, because Amazon is said to exploit its workers. Microsoft was also in the press, when a senior manager was arrested on federal charges for stealing more than 9 million USD from the company to pay for a lavish lifestyle. Steve Jobs of Apple, on the other hand, was known for an aggressive leadership style, shouting at and humiliating others (Isaacson, 2011).

It is intuitively compelling that an abusive leader, who shouts, has a different effect on a follower than a leader who exploits followers, or a leader who violates organizational rules. Unfortunately, we do not have empirical evidence to know if this is simply a lay assumption or if followers do have different reactions to different types of destructive leader behaviors. One reason for this is that comparative research in the field of destructive leadership is scarce. Rather, empirical work in the field is characterized by isolated investigations of separate destructive leadership constructs, resulting in a body of evidence that seems somewhat scattered and disconnected. This is unfortunate for both theory and practice. From a theoretical perspective, we still know too little about the unique and relative contributions of different destructive leader behaviors regarding negative follower outcomes. As a consequence, practitioners have little guidance when it comes to distinguishing, detecting, and managing different forms of destructive leadership in organizational contexts.

This is further aggravated since a broad body of research evidence suggests that negative information has a stronger influence on us and that we perceive and process negative events in a more nuanced way than positive ones (Baumeister et al., 2001; Unkelbach et al., 2008). This “bad is stronger than good” phenomenon has important implications for the domain of leadership. Not only are destructive leader behaviors likely to have a far stronger impact on followers than constructive behaviors, but the adverse impact of such destructive behaviors is likely to outweigh the benefits gained from positive relationships (e.g., with coworkers or customers). Negative interactions with a leader are likely perceived as more nuanced and more dissimilar from each other than in the case of positive information about the leader (Unkelbach et al., 2008). In our view, this makes understanding the differential effects of different destructive leader behaviors even more urgent. Thus, our main purpose in this article is to investigate whether and to what degree different types of destructive leadership may affect followers in a distinct way. In our theoretical model, we draw on the work of Einarsen et al. (2007) and Schyns and Schilling (2013). We argue that the target of the leader behavior and the level of hostility are key factors in understanding the potentially unique effects of different types of destructive leader behavior on followers. Specifically, we focus in our study on three constructs of destructive leadership: (1) abusive supervision (Tepper, 2000), as a behavior high on hostility focusing on the follower; (2) exploitative leadership (Schmid et al., 2017), as a behavior low on hostility focusing on the follower; and (3) organization-directed destructive leadership (Thoroughgood et al., 2012), as a behavior low on hostility focusing on the organization.

In order to answer the question of how far these different destructive leader behaviors elicit different reactions in followers, we draw on emotions as the first reaction to an interaction with a leader (Dasborough, 2006). Furthermore, we investigate the intention to leave, one of the most well-researched outcomes in destructive leadership research (Schyns and Schilling, 2013) and highly relevant to organizations. We thus deem these outcomes as most suited to understanding different follower reactions to destructive leadership.

## Theoretical background

Leadership is one of the most important relationships in the workplace and the way leaders give direction, assign tasks, and handle conflict has a strong influence on followers (Yukl, 2012). With this, it becomes particularly important to consider what social research refers to as “negativity bias.” In a seminal article famously titled “Bad is Stronger than Good,” Baumeister et al. (2001) cite extensive evidence showing that bad events and interactions “have more impact than good ones, and bad information is processed more thoroughly than good” (p. 323). To account for this phenomenon, Baumeister et al. (2001) draw on evolutionary selection: in order to survive threats, it was important for organisms to recognize and remember negative information more strongly than positive. As a consequence, negative information has greater emotional and motivational significance. This has important implications for the study of destructive leadership, since destructive leaders should therefore have a strong influence on followers' emotional state and their motivation to act.

Related to this, more recent research indicates that there is a significant difference between how we generally process positive versus negative information. Unkelbach et al. (2008) describe this in the density hypothesis. They argue that information is generally perceived as more similar to other positive information compared to negative information's similarity to other negative information (i.e., negative information is perceived as more dissimilar to other negative information). Thus, while destructive leadership generally impacts followers more strongly, followers may also be very sensitive to the unique features of different destructive leader behaviors.

Against this backdrop, a great deal of attention has been given to the nature and processes of destructive leadership over the last 15 years (for a review, see Schyns and Schilling, 2013). Different definitions and constructs of destructive leadership exist, all describing different behaviors. The most widely researched construct is abusive supervision (Schyns and Schilling, 2013). This refers to repeated hostile and aggressive yet nonphysical behaviors toward followers (Tepper, 2000). One of the most recent constructs describes a more prevalent form: exploitative leadership (Schmid et al., 2017) refers to genuinely self-interested leader behaviors, such as using followers for personal gain and taking credit for followers' work. Other researchers have pointed to destructive leader behaviors such as accepting bribes, stealing, or making personal use of company property (Einarsen et al., 2007; Thoroughgood et al., 2012).

In short, the literature on destructive leadership describes a multitude of different constructs (for a review, see Schyns and Schilling, 2013). At the same time, efforts have been made to integrate and organize these different approaches (Einarsen et al., 2007; Thoroughgood et al., 2012; Krasikova et al., 2013; Schyns and Schilling, 2013). In the present work, we follow the seminal taxonomy provided by Einarsen et al. (2007), who describe destructive leadership behavior along two dimensions: destructive leader behaviors targeting the followers versus destructive behaviors that target the organization. This distinction is well established and commonly used when it comes to organizing empirical evidence on destructive leadership (Aasland et al., 2010; Thoroughgood et al., 2012; Schyns and Schilling, 2013). In addition, we follow the work of Schyns and Schilling (2013), who concluded that the core of destructive leadership lies in the hostile or hindering nature of the leader's behavior. They defined destructive leadership as “a process in which over a longer period of time the activities, experiences and/or relationships of an individual or the members of a group are repeatedly influenced by their supervisor in a way that is perceived as hostile and/or obstructive” (2013, p. 141).

**Figure 1:**
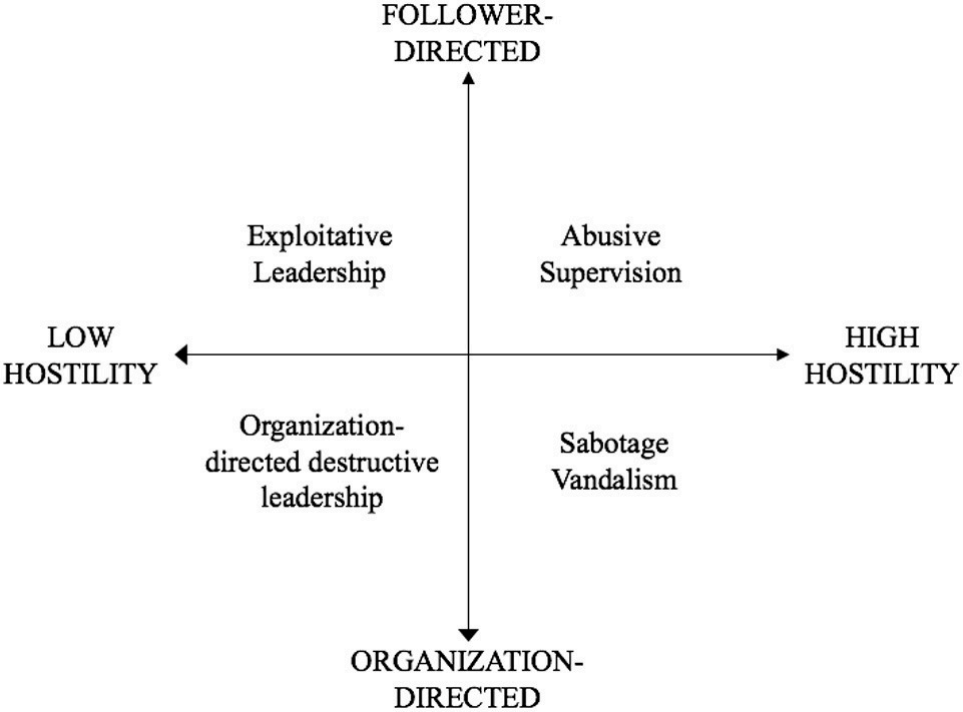
Destructive leadership types. The mentioned constructs are not exhaustive but reflect the most typical construct for each category.

Taken together, these two aspects (i.e., the target of behavior and the level of hostility) offer a useful basis for differentiating constructs. Cross-tabulation of the two dimensions results in four theoretical destructive leadership behavior categories, as shown in Figure 1. The underlying rationale for these categories is presented below.

### Follower-directed behaviors high in hostility

Constructs describing follower-directed destructive leader behaviors usually stem from the bullying literature (Tepper, 2000) and refer to genuinely abusive forms of leadership, high in hostility. The most widely researched construct appears to be abusive supervision (Schyns and Schilling, 2013). Abusive supervision refers to “subordinates' perceptions of the extent to which their supervisors engage in the sustained display of hostile verbal and nonverbal behaviors, excluding physical contact” (Tepper, 2000, p. 178). Other variants of this notion are, for instance, petty tyranny (Ashforth, 1994), social undermining (Duffy et al., 2002), strategic bullying (Ferris et al., 2007), or despotic leadership (De Hoogh and Den Hartog, 2008). While none of these constructs conceptualizes follower-directed destructive leadership in exactly the same way, they all have in common that they describe leaders who behave in a hostile and aggressive (yet nonphysical) manner toward followers. This includes repeatedly intimidating and belittling followers. However, the most established assessment of these constructs is abusive supervision (Tepper, 2000).

### Follower-directed behaviors low in hostility

Recently, Schmid et al. (2017) have introduced the concept of exploitative leadership to describe a prevalent leadership behavior that targets the followers but is not inherently hostile or aggressive. Exploitative leadership describes behaviors “with the primary intention to further the leader's self-interest by exploiting others, reflected in five dimensions: genuine egoistic behaviors, taking credit, exerting pressure, undermining development, and manipulating” (Schmid et al., 2017, p. 26). Self-interested behaviors, such as taking credit for followers' work or undermining the development of followers to benefit the leader, are low in regard to hostility. Schmid et al. (2017) posited that exploitative leadership may even be overtly friendly toward followers. Certainly, we can imagine situations where the self-interested behaviors of a leader may even benefit the organization. If a leader's goals and the organization's goals align, the leader may push followers to achieve higher targets. This may be done in a seemingly friendly way, and not by being directly abusive.

### Organization-directed behaviors low in hostility

Thoroughgood et al. (2012) described organization-directed destructive leadership around behaviors that violate the established rules and social norms of conduct in an organization. There is a broad variety of behaviors that fall under this category—for instance, theft (e.g., stealing small materials such as pens, but also money or time), talking negatively about the organization, using company properties for personal gain, as well as fraud or corruption, and even substance abuse at work (Thoroughgood et al., 2012). While these behaviors certainly vary in terms of their seriousness and harmfulness for the organization, they are not high on hostility as such.

### Organization-directed behaviors high in hostility

Behaviors that fall under the category of organization-directed leadership characterized by high levels of hostility have not been explicitly described in the destructive leadership literature. However, from a theoretical viewpoint and borrowing from research in the field of workplace deviance (Martinko et al., 2002), such behaviors refer to acts of genuine aggressiveness toward the organization. Examples would be sabotage, equipment destruction, or vandalism (e.g., spreading computer viruses). We assume that this type of destructive leadership represents a low base rate phenomenon. While this is in part true for all forms of destructive leadership, such explicitly hostile behaviors against the organization are likely to be performed particularly covertly and thus remain unseen by others. As such, they are less likely to elicit effects on followers. Thus, in the current study, we focus on those behaviors that are more prevalent and feasible to assess and that are established constructs in the destructive leadership literature.

In conclusion, we propose that two important differentiating factors of destructive leadership are: (1) the level of hostility and (2) the target of the behavior. Based on this, in the next section we develop different hypotheses for three recurring destructive leadership behaviors: abusive supervision, exploitative leadership, and organization-directed destructive leadership.

## Different effects of different destructive leadership behaviors

In this part of our article, we delineate the proposed different effects of different destructive leadership behaviors on relevant follower outcomes.

When assuming that the target of the leader's behaviors and the level of hostility are the differentiating factors between different types of destructive behavior, these two factors would naturally impact how an employee reacts. As mentioned before, negative information, such as destructive behavior of a leader, has higher emotional and motivational significance than positive information (Baumeister et al., 2001). Thus, we first assume that followers' emotions, as the most proximal reaction (Sy et al., 2005; Bono and Ilies, 2006; Bono et al., 2007) when confronted with destructive leadership, are likely to differ as a function of different destructive leadership behaviors.

Secondly, we follow the argument by Baumeister et al. (2001) that negative information has a strong motivational significance, in that it triggers an action (e.g., avoiding a negative stimulus). Thus, when relating this to destructive leadership, different levels of hostility are likely to have a different impact on the motivation to leave a leader. We thus propose to focus on emotions and the intention to leave the leader (i.e., turnover intention) in analyzing the different effects of abusive supervision, exploitative leadership, and organization-directed destructive leadership.

A very proximal effect a leader's behaviors have is on their followers' emotions (Sy et al., 2005; Bono and Ilies, 2006; see, for example, Bono et al., 2007). As such, all experiences of destructive leadership are likely paralleled by negative emotions. However, the extent of the negative affect is thought to vary, depending on the level of hostility and if the follower is targeted directly. Several scholars (e.g., Schaubhut et al., 2004; Tepper, 2007; Thau and Mitchell, 2010) have argued that destructive leadership is destructive since it is a threat to the self-worth of the followers. Abusive supervision is described as rather high on hostility. By targeting the follower—for instance, by ridiculing followers in front of others or even telling them they are incompetent—abusive supervisors would very directly harm the self-worth of followers (Burton and Hoobler, 2006). Accordingly, hostile and aggressive behaviors, such as described in abusive supervision, have been consistently related to negative affect in empirical studies (Aquino et al., 1999; Tepper, 2007). In line with this, we posit that abusive supervision has a strong impact on employees' negative affect.

Exploitative leaders, on the other hand, will take credit for work or manipulate followers to further their own self-interest. Such behaviors, while still targeting the follower directly, are lower on hostility and should thus have a less detrimental effect on followers' self-worth. While being exploited would certainly relate to negative affect, we posit that it does so less strongly than abusive supervision. On the other hand, leaders that show anti-organizational behaviors (Thoroughgood et al., 2012) will show negative behaviors that are not a direct attack on the followers' self-worth. Stealing from the organization, or talking negatively about it, primarily targets the organization, and is rather distal from the follower. We posit that this should have the least strong effect on followers' negative affect.

Thus, we specify the following predictions:

*Hypothesis 1:* All three destructive leader behaviors (i.e., abusive supervision, exploitative leadership, and organization-directed destructive leadership behaviors) will have a positive relationship with follower negative affect.
*Hypothesis 1a:* Abusive supervision will have a stronger positive relationship with followers' negative affect in comparison to exploitive leadership and organization-directed destructive leadership behaviors.
*Hypothesis 1b:* Exploitative leadership will have a stronger positive relationship with followers' negative affect in comparison to organization-directed destructive leadership behaviors.

Tepper et al. (2009) argued that when followers are confronted with self-worth threatening interactions, they feel a need to empower themselves. A very strong way to empower themselves is turnover, since a follower who intends to leave the job is less dependent on their supervisor (Tepper et al., 2009). We expect exploitative leadership, just like abusive supervision and organization-directed destructive leadership, to relate to general turnover intentions, as previous research has shown (Tepper, 2000; Schyns and Schilling, 2013; Schmid et al., 2017). We therefore predict that all three leadership styles will cause followers to reconsider their employment options. However, the degree of self-worth threat is assumed to vary depending on the level of hostility and how directly a follower is targeted by the behavior. We thus expect that the urgency of the turnover intentions will vary. Since abusive supervision represents a more direct attack on the follower with high levels of hostility, this should relate to followers considering immediate turnover (i.e., leaving the situation immediately). We argue that exploitative leadership, as a less hostile behavior, poses less of a self-worth threat to followers, resulting in a less immediate need to leave the situation. Therefore, followers under exploitative leadership will take a rather more calculative approach and consider staying until, for example, the next career level is reached. Since organization-directed destructive leadership behaviors are more distal and do not target the follower directly, the effect is more difficult to predict. It may be that a leader harming the organization confronts followers with behaviors that run against their feeling of what is right and wrong. On the other hand, the anti-organizational behavior of the leader may be too distant; as Thoroughgood et al. (2012, p. 18) put it “…such behaviors might not increase turnover intentions as quickly as overtly abusive acts.” Therefore, we only specify hypotheses for abusive supervision and exploitative leadership.

**Figure 2:**
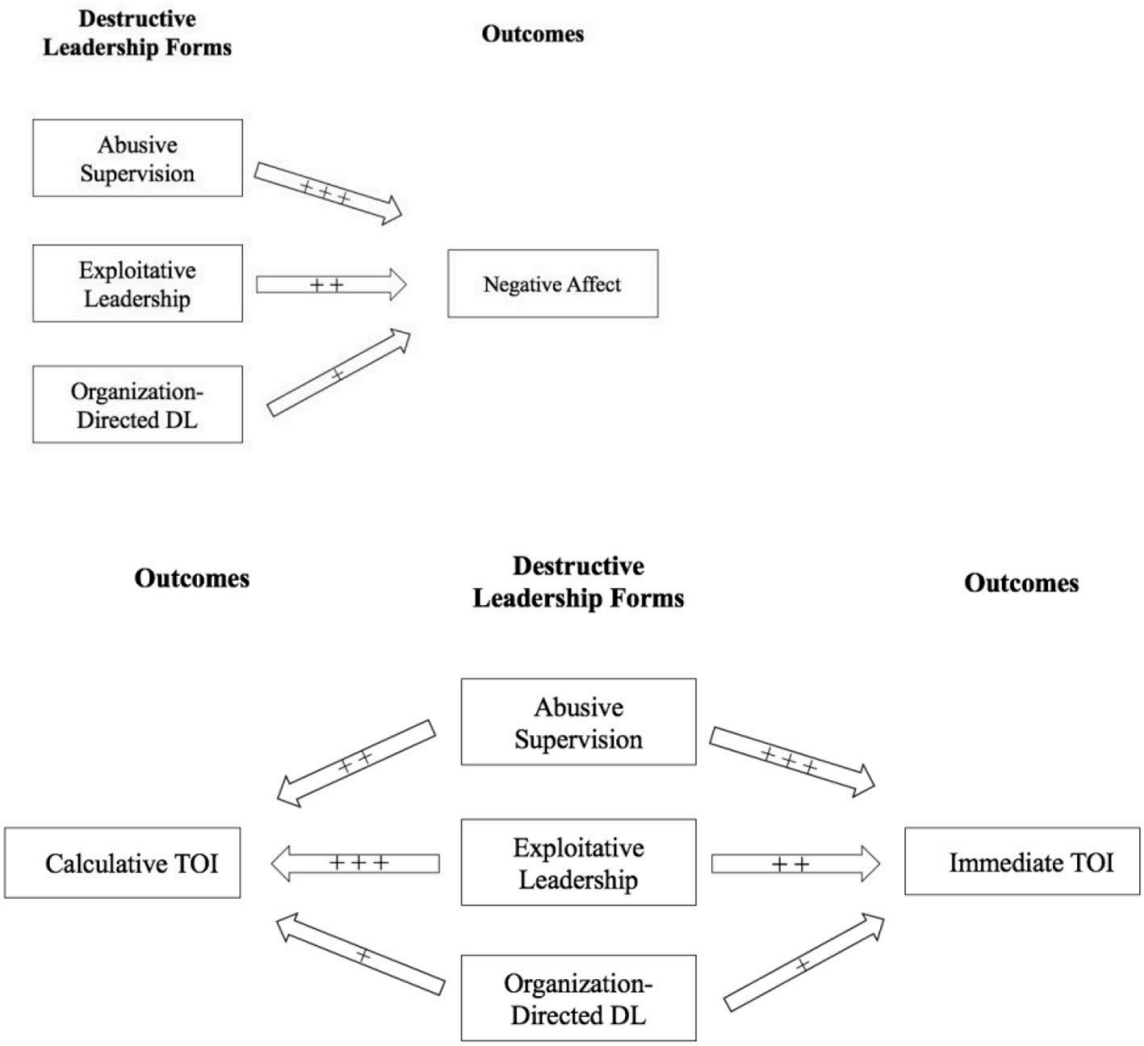
Research model. + + + indicates the strongest hypothesized effect; DL, destructive leadership; TOI, turnover intentions.

*Hypothesis 2:* All three destructive leadership behaviors (i.e., abusive supervision, exploitative leadership, organization directed destructive leadership) will have a positive relationship with general turnover intentions.
*Hypothesis 2a:* Exploitative leadership will have a stronger positive relationship with followers' calculative turnover intentions in comparison to abusive supervision and organization-directed destructive leadership behaviors.
*Hypothesis 2b:* Abusive supervision will have a stronger positive relationship with followers' immediate turnover intentions in comparison to exploitative leadership and organization-directed destructive leadership behaviors.
Our research model is shown in Figure 2.

## Methods

To test the hypotheses under investigation, we conducted two studies with different designs. In Study 1, we used a working sample and adopted a scenario-based approach to manipulate destructive leadership (i.e., abusive supervision, exploitative leadership, organization-directed destructive leadership). Then, respondents were randomly assigned to one of three conditions and provided self-reports on affective reactions and turnover intentions. Study 2 was a field study in which employees from various occupations and organizations rated their immediate supervisor in terms of destructive leadership (i.e., abusive supervision, exploitative leadership, organization-directed destructive leadership). In line with Study 1, self-reports of affective reactions and turnover intentions were collected.

We certify that the research presented in this manuscript has been conducted within the ethical standards of the DGP (German Psychological Society) regarding research with human participants and scientific integrity. We adhere to the ethical standards of the DGP, since in Germany there is no legal regulation for approval of research through a research ethics committee for the social sciences, but ethics questions are addressed within a framework by professional associations.

### Study 1

#### Sample and procedures

Building on prior research on leadership that has successfully used the vignette method (e.g., De Cremer, 2006; Van Dierendonck et al., 2014), we created three hypothetical scenarios for abusive supervision, exploitative leadership, and organization-directed destructive leadership behaviors by covering the core elements of each construct (see Appendix).

Participants for this study were recruited via an open online survey conducted within the network of three Master's students. On the first page of the online survey, participants were informed that participation was voluntary and by continuing to the second page, they consented to participating in the study. A prerequisite for participating in the survey was that participants were employed full time. In total, 297 participants took part in the online survey and were randomly assigned to one of the three experimental groups (92 in the exploitative leadership, 113 in the abusive supervision, and 92 in the organization-directed destructive leadership condition). In total, 136 respondents were female, the mean age was 25.64 (*SD* = 7.04), and the majority of the participants (95.6 percent) worked in the for-profit sector.

#### Measures

##### Manipulation check

After presenting respondents with the scenarios, they were asked to rate them in terms of abusive supervision, exploitative leadership, and organization-directed destructive leader behaviors to test whether the manipulation of the independent variable was successful. Exploitative leadership was assessed by six items taken from the exploitative leadership scale (α = 0.87) introduced by Schmid et al. (2017). These six items covered the five dimensions of exploitative leadership (i.e., egoism, taking credit, exerting pressure, undermining development, manipulating). An example item was “This leader prioritizes their own goals over the goals and needs of followers.” Abusive supervision was measured by six items taken from the abusive supervision scale by Tepper (2000; α = 0.89). An example item was “This leader puts me down in front of others.” Organization-directed destructive leadership behaviors were captured with seven items from the anti-organizational leader behavior sub-scale developed by Thoroughgood et al. (2012; α = 0.92); a sample item was “This leader violates company policy/rules.” All leadership items were rated on a five-point scale (ranging from 1 = strongly disagree to 5 = strongly agree).

##### Emotional reactions

Emotional reactions Emotional reactions were measured by using the German version (Krohne et al., 1996) of the 20-item Positive and Negative Affect Schedule (PANAS; Watson et al., 1988). The PANAS contains two ten-item sub-scales to measure both negative and positive affect. In the current study, both sub-scales showed sufficient reliability (α = 0.75 for both sub-scales). Respondents were instructed to indicate the extent to which they felt this way (e.g., active, interested, or excited for positive affect versus distressed, upset, or guilty for negative affect) toward the leader described in the scenario. Responses were given on a five-point scale (ranging from 1 = very slightly or not at all to 5 = extremely).

##### Turnover intentions

We assessed three indicators related to turnover intention. Firstly, we adapted two items from Kirchmeyer and Bullin (1997) to assess general turnover intentions *(“I would start looking for a new job”*) as well as immediate turnover intentions (“I would hand in my notice immediately”). Moreover, we developed an item to measure calculative turnover intentions (“I would wait for the next career step is reached before leaving”). Responses were anchored on a five-point continuum (ranging from 1 = strongly disagree to 5 = strongly agree).

#### Results

##### Manipulation check

The manipulation check was tested by a one-way analysis of variance (ANOVA), including post-hoc comparisons using the Tukey HSD test. The results revealed a significant effect of leadership style manipulation on the perception of exploitative leadership [*F*_(2, 235)_ = 7.41, *p* < 0.001]. Post-hoc comparisons indicated that the exploitative leadership manipulation was indeed perceived as being more exploitative (*M* = 4.32; *SD* = 0.76) compared to abusive supervision (*M* = 3.94; *SD* = 0.80) and organization-directed destructive leadership behaviors (*M* = 3.87; *SD* = 0.75). Similarly, there was a significant effect of leadership manipulation on the perception of abusive supervision [*F*_(2, 237)_ = 109.33, *p* < 0.001]. *Post-hoc* analysis indicated that the abusive supervision vignette was indeed perceived as being more abusive (*M* = 4.44; *SD* = 0.64) than the exploitative leadership condition (*M* = 3.02; *SD* = 0.83) and the organization-directed destructive leadership condition (*M* = 3.00; *SD* = 0.75). Finally, we found a significant effect of leadership manipulation on the perception of organization-directed destructive leadership behaviors [*F* (2, 238) = 109.66, *p* < 0.001]. Post-hoc analysis showed that the organization-directed destructive leadership behaviors condition was indeed perceived as being more organization-directed destructive (*M* = 4.10; *SD* = 0.78) than the abusive supervision condition (*M* = 2.37; *SD* = 0.80) and the exploitative leadership condition (*M* = 2.40; *SD* = 0.84). Taken together, this pattern shows that the leadership manipulations were successful.

##### Hypothesis tests concerning followers' emotional reactions

Next, we tested our hypotheses regarding the proposed different effects of the three destructive leader behaviors. The first set of hypotheses refers to affective reactions. Although the focus of our analysis was the effects of destructive leader behavior on negative affect, we deemed it useful to account, too, for the effect on positive affect. The mean scores pertaining to the three conditions are shown in Table 1.

**Table 1:** Mean scores of emotional reactions (Study1).

	**Negative affect**	**Positive affect**
Exploitative leadership	3.14	2.56
Abusive supervision	3.74	2.38
Organization-directed destructive leadership	3.39	2.60

A multivariate analysis of variance (MANOVA) with destructive leadership as independent variable and negative and positive affect as dependent variables showed a significant multivariate effect [*F*_(4, 504)_ = 7.20, *p* < 0.001; Wilk's Λ = .89, η^2^ = 0.05]. Yet, univariate testing found the effect to be significant only for negative affect [*F*_(2, 253)_ = 13.28, *p* < 0.001], and no significant effect was found for positive affect [*F*_(2, 253)_ = 2.92, *p* = 0.06]. Post-hoc comparisons using the Tukey HSD test indicated that negative affect was significantly higher in the abusive supervision condition (*M* = 3.60, *SD* = 0.66), as compared to the exploitative leadership condition (*M* = 3.11, *SD* = 0.54) and the organization-directed destructive leadership condition (*M* = 3.34, *SD* = 0.62). No difference between the exploitative leadership and the organization-directed destructive leadership conditions was revealed. Thus, hypothesis 1 was supported.

Next, we adopted an explorative perspective and examined whether the different types of destructive leadership under investigation would be related to specific facets of negative affect. Specifically, building on the work of Mehrabian (1997), Janke and Glöckner-Rist (2014) found evidence that the negative affect items of the PANAS reflect two sub-dimensions, upset and afraid. The upset dimension contains the upset, hostile, and irritable items, whereas the afraid dimension includes the guilty, ashamed, afraid, nervous, jittery, distressed, and scared items. Using the Pleasure-Arousal-Dominance (PAD) emotion model (Mehrabian, 1996) as a framework, Mehrabian (1997) found that the upset dimension is characterized by high levels of displeasure (i.e., genuine negative emotional state) and, though less heavily, by arousal (i.e., mental and/or physical activity level). In contrast, the afraid dimension relates less strongly to displeasure, more to arousal, and also more to submissiveness (i.e., lack of control over others or situations).

The mean scores for the two sub-dimensions that we obtained in the current study are shown in Table 2. Again, a MANOVA with destructive leadership as the independent variable and the upset and afraid dimensions as dependent variables revealed a significant multivariate effect [*F*_(4, 504)_ = 15.08, *p* < 0.001; Wilk's Λ = 0.80, η^2^ = 0.11].

**Table 2:** Mean scores of negative affect sub-dimensions (Study1).

	**Upset dimension**	**Afraid dimension**
Exploitative leadership	4.16	2.67
Abusive supervision	4.25	3.19
Organization-directed destructive leadership	3.73	3.18

Separate one-way ANOVAs for each dimension showed the following pattern. For the upset dimension, we found a significant effect of the leadership manipulation [*F*_(2, 252)_ = 10.62, *p* < 0.001]. *Post-hoc* analyses revealed no significant difference between the exploitative leadership condition (*M* = 4.16, *SD* = 0.79) and the abusive supervision condition (*M* = 4. 25, *SD* = 0.72). Yet, both conditions were significantly different from the organization-directed destructive leadership condition (*M* = 3.73, *SD* = 0.81). Next, also for the afraid dimension, we found a significant main effect [*F*_(2, 253)_ = 17.20, *p* < 0.001]. Respondents scored similarly high in the abusive supervision (*M* = 3. 32, *SD* = 0.85) and the organization-directed destructive leadership conditions (*M* = 3.18, *SD* = 0.69), which were both significantly different from the exploitative leadership condition (*M* = 2. 67, *SD* = 0.63).

##### Hypothesis tests concerning followers' turnover intentions

The MANOVA we conducted showed a statistically significant difference in turnover intentions based on the leadership manipulation [*F*_(6, 482)_ = 2.69, *p* < 0.05, Wilk's Λ = 0.93, η^2^ = 0.03]. Separate ANOVAs showed the following pattern. For general turnover, we found a significant effect of the leadership manipulation [*F*_(2, 243)_ = 3.70, *p* < 0.05]. *Post-hoc* analysis using the Tukey HSD procedure revealed a significant difference between the abusive supervision (*M* = 4.52, *SD* = 0.68) and the organization-directed destructive leadership conditions (*M* = 4.24, *SD* = 0.84). For the other combinations, no significant differences were revealed. Overall, general turnover intentions were substantially high in all three conditions, thus confirming hypothesis 2. Next, for calculative turnover intentions, we found no significant effect of the leadership manipulation [*F*_(2, 243)_ = 1.24, *p* = 0.32]. Therefore, hypothesis 2a was not confirmed. Finally, immediate turnover significantly differed between the conditions [*F*_(2, 243)_ = 3.58, *p* < 0.05]. *Post-hoc* analyses showed that immediate turnover was lower in the exploitative leadership (*M* = 2.01, *SD* = 0.90) than in the abusive supervision (*M* = 2.39, *SD* = 0.92) condition, thus confirming hypothesis 2b. The organization-directed destructive leadership condition (*M* = 2.30, *SD* = 0.96) did not significantly differ from the other two groups.

#### Brief discussion

This study revealed a series of distinct effects, in particular for exploitative leadership and abusive supervision. As predicted, abusive supervision emerges as the strongest precursor to overall negative affect.

Both abusive supervision and exploitative leadership are associated with stronger feelings of displeasure (i.e., upset) compared to organization-directed destructive behaviors. Yet, with regard to the afraid dimension of the PANAS, an interesting difference was revealed, with lower scores for exploitative leadership relative to the abusive supervision condition. This suggests that abusive supervision is more strongly related to anxiety among followers, reflected in increased arousal and feelings of submissiveness (Mehrabian, 1997). With regard to turnover, all three forms of negative leadership were related to high general turnover intention. While the level of calculative turnover intention was inconspicuous among the three conditions, abusive supervision tends to relate to higher immediate turnover reactions.

Overall, the results were only partly as expected. This may be because of the hypothetical nature of the scenarios. Therefore, in Study 2 we designed a field study to test the same hypotheses.

### Study 2

#### Sample and procedures

We gathered valid responses from 167 employees from various organizations in Germany who rated their immediate leaders in terms of destructive leadership and provided self-reports on emotional reactions and turnover intentions. Respondents were contacted via snowball sampling, starting with the authors' professional network. The majority of the participants (72 percent) worked in the for-profit sector (28 percent worked in non-profit organizations or in the public sector). The mean age was 36.22 years (*SD* = 12.13) and 63.30 percent of the respondents were male. On average, the respondents had been working for their current supervisor for 4.89 years (*SD* = 5.48) and organizational tenure was 7.98 years on average (*SD* = 8.69). In terms of education, 67 percent of the respondents held a university degree.

#### Measures

##### Destructive leadership measures

Exploitative leadership was assessed with the full 15-item exploitative leadership scale developed by Schmid et al. (2017). Abusive supervision was measured according to the full 15-item abusive supervision scale by Tepper (2000). Organization-directed destructive leader behaviors were captured with the measure developed by Thoroughgood et al. (2012). Sample items can be seen in the description of measures in Study 1. Respondents rated the frequency of destructive leader behaviors on a five-point scale ranging from 1 (“not at all”) to 5 (“frequently if not always”).

##### Outcome measures

For the outcomes (i.e., emotions and turnover), we used the same items with the same response format as in Study 1.

#### Results

##### Validity of measures

Table 3 reports the descriptive statistics and correlations among the study variables.

**Table 3:** Mean scores of turnover intentions (Study 1).

	**General turnover**	**Calculative turnover**	**Immediate turnover**
Exploitative leadership	4.27	3.05	2.01
Abusive supervision	4.52	3.09	2.39
Organization-directed destructive leadership	4.24	3.30	2.30

Prior to testing the hypotheses under investigation, we examined whether the measures we used represented valid tools to assess our target constructs. To this end, we used confirmatory factor analysis (CFA) in AMOS and tested the factorial integrity of our measures. In a first step, we conducted CFA on the item level for each measure separately (i.e., exploitative leadership, abusive supervision, organization-directed destructive leadership) and examined the factor loadings and item reliabilities. While all items of the exploitative leadership measure had excellent psychometric properties, we dropped several items of the other two measures (i.e., abusive supervision, organization-directed destructive leadership) because they did not represent the underlying construct well (i.e., factor loadings were below 0.60 and item reliabilities below 0.40; Hair et al., 2006).

Next, we tested the discriminant validity of our measures. Because of the relatively large number of estimated parameters in the overall model and the small sample size, we created item parcels for all latent leadership constructs (Landis et al., 2000). For exploitative leadership, we formed five parcels based on the five dimensions specified by Schmid et al. (2017) (i.e., egoism, taking credit, exerting pressure, undermining development, and manipulation). For abusive supervision and organization-directed destructive leadership, we used the factorial algorithm to create parcels (see Matsunaga, 2008). By sequentially including the items with the highest to the lowest factor loadings, while alternating the direction of item selection, three parcels were formed for abusive supervision and two parcels for organization-directed destructive leadership.

On this basis, we tested a series of theoretically viable factor models. Table 4 shows that a three-factor model with the three target constructs as latent variables and parcels as indicators obtained the best model fit and was preferable over alternative solutions. These results provide evidence that our measures captured distinct constructs versus common source effects.

**Table 4:** Measurement models (Study 2).

**Model**	**χ^2^**	***df***	**χ^2^/*df***	**CFI**	**RMSEA**	**Δχ^2^_(df)_**
*Model 1* (3-factor model: exploitative leadership, abusive supervision, and organization-directed destructive leadership as separate factors)	79.65^***^	32	2.48	0.94	0.09	
*Model 2* (2-factor model: exploitative leadership and abusive supervision as combined factor)	151.93^***^	34	4.46	0.87	0.14	72.28${}_{\left(2\right)}^{***}$
*Model 3* (2-factor model::exploitative leadership and organization-directed destructive leadership as combined factor)	134.97^***^	34	3.97	0.88	0.13	55.32${}_{\left(2\right)}^{***}$
*Model 3* (2-factor model: abusive supervision and organization-directed destructive leadership as combined factor)	150.86^***^	34	4.43	0.87	0.14	71.21${}_{\left(2\right)}^{***}$
*Model 4* (single factor model)	206.82^***^	35	5.90	0.81	0.17	127.17${}_{\left(3\right)}^{***}$

Δχ^2^ represents the difference in χ^2^ values between the respective model and Model 1 (i.e., the proposed 3-factor model);

*** *p < 0.001*.

##### Hypothesis tests concerning followers' emotional reactions

To test our hypotheses, we conducted a series of multiple regression analyses. In addition, given the high correlations among the destructive leadership measures, we followed the procedures suggested by Lorenzo-Seva et al. (2010) and applied relative weight analysis. The results of these procedures are depicted in Tables 5, 6.

**Table 5:** Descriptive statistics and correlations (Study 2).

		**M**	**SD**	**1**	**2**	**3**	**4**	**5**	**6**	**7**	**8**	**9**	**10**
1.	Exploitative leadership	2.07	0.91	(0.95)									
2.	Abusive supervision	1.51	0.62	0.75^**^	(0.87)								
3.	Organization-directed destructive leadership	1.39	0.66	0.61^**^	0.52^**^	(0.86)							
4.	Negative affect	1.88	0.72	0.72^**^	0.77^**^	0.52^**^	(0.89)						
5.	Positive affect	3.33	0.78	−0.44^**^	−0.43^**^	−0.23^**^	−0.39^**^	(0.88)					
6.	Negative affect: upset	2.11	1.01	0.72^**^	0.75^**^	0.51^**^	0.88^**^	−0.47^**^	(0.87)				
7.	Negative affect: afraid	1.78	0.68	0.63^**^	0.68^**^	0.47^**^	0.95^**^	−0.29^**^	0.69^**^	(0.84)			
8.	General turnover	2.63	1.35	0.53^**^	0.43^**^	0.33^**^	0.47^**^	−0.39^**^	0.55^**^	0.35^**^	(–)		
9.	Calculative turnover	2.67	1.42	0.24^**^	0.01	0.18^*^	0.17^*^	−0.11	0.14	0.17^*^	0.44^**^	(–)	
10.	Immediate turnover	1.63	1.08	0.64^**^	0.54^**^	0.48^**^	0.60^**^	−0.41^**^	0.60^**^	0.52^**^	0.65^**^	0.24^**^	(–)

** p < 0.01;

* *p < 0.05; Cronbach's alpha appears on the diagonal*.

**Table 6:** Effects of destructive leadership on overall negative and positive affect (Study 2).

**Predictors**	**Beta**	**Relative weights**	**95% Confidence interval**	
			**LL**	**UL**
**OUTCOME: OVERALL NEGATIVE AFFECT**				
Exploitative leadership	0.29^***^	32.40	26.10	39.70
Abusive supervision	0.50^***^	51.00	42.00	60.40
Organization-directed destructive leadership	0.08	16.60	9.30	27.20
*R^2^*	0.64			
*F*	98.77^***^			
**OUTCOME: OVERALL POSITIVE AFFECT**				
Exploitative leadership	−0.27^**^	44.00	26.40	60.60
Abusive supervision	−0.29^*^	46.40	27.10	64.10
Organization-directed destructive leadership	0.09	9.60	6.80	21.50
*R^2^*	0.21			
*F*	15.65^***^			

*** p < 0.001;

** *p < 0.0; relative weights reflect the relative contribution to R^2^ (percentages)*.

Abusive supervision was the strongest predictor of overall negative affect (β = 0.50, *p* < 0.001), followed by exploitative leadership (β = 0.29, *p* < 0.001), and organization-directed destructive leadership (β = 0.08, *ns*). Thus, hypothesis 1 was supported. For overall positive affect, abusive supervision (β = −0.27, *p* < 0.01) and exploitative leadership (β = −0.29, *p* < 0.05) exerted a similar negative effect, while the effect for organization-directed destructive leadership was not significant (β = 0.09, *ns*). With regard to the sub-dimensions of negative affect (see Table 7), the following pattern was revealed: the upset dimension was best predicted by abusive supervision (β = 0.47, *p* < 0.001), followed by exploitative leadership (β = 0.33, *p* < 0.001). The effect for organization-directed destructive leadership was not significant (β = 0.06, *ns*). In a similar vein, abusive supervision was the strongest predictor for the afraid dimension (β = 0.46, *p* < 0.001) followed by exploitative leadership (β = 0.23, *p* < 0.05). Again, organization-directed destructive leadership had no predictive value here (β = 0.09, *ns*).

**Table 7:** Effects of destructive leadership on negative affect sub-dimensions (Study 2).

**Predictors**	**Beta**	**Relative weights**	**95% Confidence interval**	
			**LL**	**UL**
**OUTCOME: NEGATIVE AFFECT (UPSET)**				
Exploitative leadership	0.33^***^	34.40	26.20	41.80
Abusive supervision	0.47^***^	49.70	41.80	58.00
Organization-directed destructive leadership	0.06	15.90	9.30	25.20
*R^2^*	0.63			
*F*	93.89^***^			
**OUTCOME: NEGATIVE AFFECT (AFRAID)**				
Exploitative leadership	0.23^*^	31.00	23.60	40.40
Abusive supervision	0.46^***^	51.90	40.00	62.20
Organization-directed destructive leadership	0.09	17.10	8.50	30.10
*R^2^*	0.50			
*F*	55.57^***^			

*** p < 0.001;

** *p < 0.0; relative weights reflect the relative contribution to R^2^ (percentages)*.

The next set of hypotheses refers to different types of turnover intention. For general turnover intention, the results of regression analysis revealed only exploitative leadership as a significant predictor (β = 0.47, *p* < 0.001). Relative weight analysis, however, showed that the other two leadership forms also explained variance in general turnover intention (see Table 8); however, exploitative leadership clearly exerted the strongest effect. While these results do not fully confirm hypothesis 2, relative weights analysis does point to an effect in the expected direction.

**Table 8:** Effects of destructive leadership on turnover intentions (Study 2).

**Predictors**	**Beta**	**Relative weights**	**95% Confidence interval**	
			**LL**	**UL**
**OUTCOME: GENERAL TURNOVER INTENTIONS**				
Exploitative leadership	0.47^***^	54.00	38.30	66.50
Abusive supervision	0.07	30.30	18.20	45.40
Organization-directed destructive leadership	0.01	15.70	8.10	29.40
*R^2^*	0.28			
*F*	22.39^***^			
**OUTCOME: CALCULATIVE TURNOVER INTENTIONS**				
Exploitative leadership	0.48^***^	51.30	28.00	67.30
Abusive supervision	−0.39^***^	29.60	16.30	46.30
Organization-directed destructive leadership	0.10	19.10	7.70	46.00
*R^2^*	0.11			
*F*	7.93^***^			
**OUTCOME: IMMEDIATE TURNOVER INTENTIONS**				
Exploitative leadership	0.46^***^	45.00	33.20	58.20
Abusive supervision	0.13	30.90	17.70	44.50
Organization-directed destructive leadership	0.13	24.10	12.10	39.20
*R^2^*	0.42			
*F*	41.23^***^			

*** *p < 0.001; relative weights reflect the relative contribution to R^2^ (percentages)*.

With regard to calculative turnover, we found a positive effect for exploitative leadership (β = 0.48, *p* < 0.001), whereas the effect of abusive supervision was negative (β = −0.39, *p* < 0.001). Given that the two predictor variables were highly correlated (*r* = 0.75, *p* < 0.001), while abusive supervision did not correlate with the outcome variable (*r* = 0.01, *ns*), this pattern shows the classic signs of a suppression effect (Tzelgov and Henik, 1991). This means that abusive supervision shares no or only little variance directly with the outcome variable but contributes to the regression equation by removing irrelevant variance from the other predictor variables. This is also reflected in the results of relative weight analysis, showing that exploitative leadership explained the major portion of variance in calculative turnover intentions (see Table 6). While hypothesis 2a is again not fully confirmed, taken together, this pattern points to what was predicted.

Interestingly, for immediate turnover, only exploitative leadership was a significant predictor in the regression analysis (β = 0.46, *p* < 0.001). Again, relative weight analysis revealed that the other two leadership forms also explained variance in immediate turnover intention, yet only to a moderate extent (see Table 6). Thus, hypothesis 2b was not supported.

#### Brief discussion

In line with the results found in Study 1, both abusive supervision and exploitative leadership were found to have a negative relationship with positive affect. With regard to negative affect, however, different patterns were found. Abusive supervision was related most strongly to overall negative affect and to the afraid sub-dimension of negative affect. Also, it was more strongly related to the upset sub-dimension, relative to exploitative leader behavior. This is different from what we found in Study 1, where exploitative leadership had an equally strong effect on the upset sub-dimension. Overall, the pattern found in Study 2 supports the notion that abusive supervision is both generally and relative to exploitative leadership more strongly related to negative emotional reactions of followers. Organization-directed destructive leader behavior seems to play a marginal role when it comes to followers' emotional reactions. A potential explanation for this could be that followers perceive such leader behaviors as rather distal—i.e., as actions they can more efficiently distance themselves from.

For turnover intentions, the results are more complex. While all three types of destructive leadership behavior relate to general turnover intention, when examining the relative weights, exploitative leadership has the strongest relationship. However, exploitative leadership had the strongest positive relationship with calculative turnover intention—i.e., followers would stay until the next milestone in their career was reached before leaving—whereas abusive supervision had limited impact. This is in line with our hypothesis: because of the stronger self-worth threat, followers would be less likely to have a calculative approach.

However, when looking at immediate turnover intention, a low effect was found for abusive supervision. Whereas this may seem counterintuitive at first, the underlying explanation may be that the decision to leave a job depends on many factors that are situational, and may depend on the individual follower's personality.

## General discussion

The main purpose of this article was to investigate if different destructive leadership behaviors may affect followers in a distinct way. Our focus was on three destructive leadership constructs: abusive supervision (Tepper, 2000), exploitative leadership (Schmid et al., 2017), and organization-directed destructive leadership (Thoroughgood et al., 2012). To answer the question of how far these behaviors would elicit different reactions in followers, we investigated followers' emotions as the first reaction to an interaction with leaders (Dasborough, 2006) and the intention to leave. The results of both a scenario-based experimental study and a field study suggest that exploitative leadership does indeed influence different outcomes compared to leaders behaving in an abusive manner or leaders behaving in a manner that harms the organization. As expected, all three constructs had a positive relationship with negative affect. Yet, with regard to the afraid dimension of the PANAS, higher scores for abusive supervision were found. Organization-directed destructive leader behavior showed marginal relevance with regard to different urgencies of turnover intention. However, exploitative leadership and abusive supervision affected calculative and immediate turnover intentions to a similar degree. In what follows, we discuss the theoretical and practical implications of these results in more detail.

### Theoretical implications

In line with prior research (Schyns and Schilling, 2013), our results confirm that destructive leadership is a critical source of negative affect among followers. Since negative affect has generally been shown to undermine employees' social wellbeing and productivity (Barsade and Gibson, 2007; Elfenbein, 2007; for overviews, see Ashkanasy and Dorris, 2017), it is theoretically and practically useful to understand the different influence leaders may have in this regard. Our results extend existing knowledge by showing that different forms of destructive leader behavior have different effects on both the type and intensity of negative affect. Specifically, our results indicate that, in contrast to exploitative leadership, abusive supervision is more strongly related to anxiety among followers. This anxiety is reflected in increased arousal and feelings of loss of control (Mehrabian, 1997). These higher levels of anxiety may be explained through the more hostile and direct attack on the follower posed by abusive supervision. This hostility—i.e., shouting at followers or ridiculing them in public—is a high threat to the self-worth of the follower (e.g., Schaubhut et al., 2004; Tepper, 2007; Thau and Mitchell, 2010) and may thus results in feelings of submissiveness and anxiousness.

This difference in follower emotional reactions is important, since previous research indicates that negative affect is related to stronger effects in organizations than positive affect, and also to more nuanced effects on followers' behavior (e.g., Baumeister et al., 2001; Dasborough, 2006). As an example, previous research suggests that different emotions relate to how employees' attribute blame (Gooty et al., 2009). Related to our results, this means that anxiety in followers may likely be related to blame being attributed internally, and so followers blaming themselves for leaders' behavior. Thus, abusive supervision may be more likely to result in internal attributions of blame, whereas followers with an exploitative leader may rather attribute blame externally—i.e., blame the leader for taking credit for their work. This attribution will likely set in motion very distinct behavioral dynamics, since whether employees attribute destructive leadership internally or externally has been linked to the occurrence of distinct forms of workplace deviance. Specifically, according to the causal reasoning model of counterproductive work behavior (Martinko et al., 2002), external attributions are more likely to trigger retaliatory behaviors (such as hiding knowledge or sabotage), whereas internal attributions are thought to trigger more self-destructive deviance (such as drug and alcohol abuse; Bamberger and Bacharach, 2006). Thus, the difference in attribution relates to very different follower behaviors (Gooty et al., 2009); previous research has shown, furthermore, that it also relates to decision-making and risk-taking (Forgas and George, 2001). Thus, an abusive supervisor, by relating to higher anxiety in followers, may inhibit risk-taking behavior which would in the long run impede the innovation and flexibility of teams and ultimately the entire organization.

Although our results relate to emotions at the individual follower level, they nevertheless imply that different destructive leader behaviors trigger distinct emotional reactions in followers, and these different emotional reactions may trigger very distinct dynamics in teams and organizations. In fact, emotions in organizations are described as a multilevel phenomenon and Ashkanasy and Dorris (2017) posited that emotions act at different levels ranging from the individual level to the team level and the organizational level. Leadership plays an important role in this multilevel phenomenon, since it enables emotions to spread from the individual to the organization through the process of emotional contagion (Hatfield et al., 1992).

A similar pattern, suggesting distinct dynamics resulting from different destructive leader behaviors, was found for turnover intention. Although our results regarding immediate and calculative turnover intention are not as clear as expected, they do suggest that different leader behaviors, depending on how hostile they are and how directly they attack the follower, may trigger a more or less immediate need to act. Thus, the time frame for different destructive leader behaviors to unfold their destructive effect may vary. In a related line of research, it was shown that narcissists make good first impressions and only over time, when their dark side shows, do perceptions others have of them change for the worse (Paulhus, 1998). Narcissists are seen as charming and confident on first encounter, while their exploitative and manipulative side only shows over time, leading to a delay in negative effect on others. A similar effect can be assumed for exploitative leadership. Schmid et al. (2017) described that exploitative leadership can be seemingly friendly. The hostile behaviors of an abusive leader may be more immediately threatening and harder to tolerate on a daily basis.

However, our results also show a counterintuitive pattern: that is, in Study 2, followers' intentions to immediately leave an abusive leader were low. The underlying explanation may be that the decision to leave a job depends on many factors, and we need to consider situational as well as individual factors. An important factor is the availability of other employment options. Thus, the socioeconomic environment needs to be taken into account. Besides the job market, another important factor is how a follower judges their employability. Victims of abuse are often low in self-esteem (see, for example, Aguilar and Nightingale, 1994) and may not rate their employability very highly; they may thus remain in an (abusive) workplace, although it seems counterintuitive.

As mentioned above, attribution may play an important role in unfolding the destructive leadership dynamic (Gooty et al., 2009). While emotions relate to different attribution patterns, followers' individual attribution style should also play an important role. Different attribution of why the leader is showing certain destructive behaviors will relate to different conclusions and, in consequence, different follower behaviors (see, for example, Peus et al., 2012). An abusive supervisor, showing hostile behaviors, may rather lead to an attribution of hostile intentions, whereas an exploitative leader, taking credit for others' work and manipulating others to advance their career, may be seen as rather overly ambitious. While this will naturally lead to different individual follower behavioral reactions, we can also imagine that it will impact the team dynamics differentially. Whereas a leader that is seen as hostile may prompt a team to rally together and create cohesion, a leader that is exploitative may rather create a focus on individual self-interest in the team (Peus et al., 2012).

Taken together, our results show a very complex pattern of different destructive leader behaviors and point to the importance of understanding nuances in destructive leadership. Since previous research suggests that it may be easier to discourage desired follower behaviors, such as creativity, than to encourage them (e.g., Kark et al., 2018), understanding how the destructive leadership dynamics unfold seems crucial for organizations. With this study, we contribute to the advancement of destructive leadership theory and methodology by providing empirical evidence that followers indeed have different reactions to different destructive leadership behaviors and that these reactions are able to provide unique information in terms of predicting followers' emotions and turnover intentions. This has important implications for the landscape of destructive leadership, since the literature so far has overlooked important insights from a methodological, theoretical, and practical perspective. From the perspective of theory advancement, we may overlook mediators and outcomes that are specific to a certain type of destructive leadership behavior (Herschcovis and Barling, 2010). From a methodological perspective, the fact that the majority of studies examine one type of destructive leadership in relation to an outcome (Schyns and Schilling, 2013) and do not compare the effects of different destructive leadership behaviors may result in under- or over-estimations of the true effects (Herschcovis and Barling, 2010). Related to this, with this being only the second empirical study on exploitative leadership that we are aware of, we also make a further contribution to the construct validity of the new construct of exploitative leadership (Schmid et al., 2017).

### Practical implications

Knowing that different kinds of destructive leadership impact followers differently has important implications for practice. Practitioners, for the purpose of leadership development and coaching, will be able to understand destructive leadership in a more nuanced manner. This allows for more tailored interventions that take into account the impact that is likely to be expected from a certain type of destructive behavior. Related to this, in our view, the results of our studies generally point to the importance of customizing organizational interventions. This means that first the destructive leader behavior needs to be assessed to understand it in terms of the target of the behavior and the level of hostility. Next, interventions can be chosen—for example, personal coaching for the leader can work on the specific harming behaviors. In targeting specific behaviors in a customized way in coaching and training, digital learning methods can be highly beneficial in offering individualized solutions. For instance, apps are used to help leaders apply new behaviors in their daily work and receive instant feedback. With knowledge about the specific behaviors, mechanisms, and effects of different types of destructive leader behavior, this may be a promising avenue for future leader training on the job.

### Limitations and future research

Despite the contributions, our studies are not without their limitations. In Study 1, we chose an experimental vignette approach, which naturally has a range of limitations. Scenarios, rather than real experiences, may reflect the perception of participants. Whereas internal validity is high in our scenario study, the generalizability is limited. Nevertheless, it has been shown that scenario experiments tend to score well on common realism (Van Dierendonck et al., 2014). In Study 2, we further conducted a field study to gain an understanding of real organizational effects. However, this was a measurement at one point in time and relied solely on followers' perceptions, thus being prone to common method bias. While self-reports are certainly well-suited to capture followers' emotional reactions and individual attitudes (Conway and Lance, 2010), future research in this field may benefit from using more objective measures, such as physiological reactions (Mauss and Robinson, 2009). A further limitation refers to our use of single-item measures for the different facets of turnover intention, most notably with regard to measurement reliability. Yet, prior research has demonstrated that single-item measures are a reasonable option under certain circumstances (e.g., Fisher et al., 2016). On the one hand, we chose single-item measures to minimize respondent burden while increasing face validity. Moreover, we consider the facets of turnover intention rather concrete and specific, so that a general single item enhances respondents' clarity regarding what is actually being measured (Fuchs and Diamantopoulos, 2009; Fisher et al., 2016). It would certainly be fruitful in future research to test multiple- item measures to capture different facets of turnover intention.

Overall, our research opens up multiple avenues for future research. While we have focused on two types of outcome, there are certainly many more outcomes and important mechanisms that would benefit from a more differentiated view. In our view, the most promising next route would be to investigate mechanisms that can shed further light on how different types of destructive leader behavior influence followers. Organizational justice theory has been studied as an important mechanism for destructive leadership (Tepper et al., 2006) and we can imagine that the different types of organizational justice may work as mechanisms with different types of destructive behavior. Whereas abusive supervision may more strongly relate to perceptions of interpersonal unfairness, exploitative leadership will rather violate concerns of distributive and procedural justice. In a similar vein, our results suggest that negative affect may not even be the primary mechanism through which exploitative leadership affects followers. Rather, in contrast to abusive supervision, with its strong focus on hostility and aggression, exploitative leadership may work more strongly through follower cognition than affect. Future research should test this assumption by considering follower outcomes that are inherently cognitive, such as reciprocity expectations (Bernerth et al., 2007).

Furthermore, qualitative studies would be of great interest to shed light on the differences in perceptions and effects of leaders behaving destructively in either an exploitative, an abusive, or an organization-directed way. Specifically, qualitative interviews are especially suited to examine mechanisms and reasons why followers react in certain ways to destructive leadership.

Of interest, furthermore, would be to investigate different effects of different destructive leadership behaviors in a long-term field study, to capture real and longer-term follower-leader interactions. We would argue that destructive behaviors high on hostility, such as abusive supervision, would lead to negative effects on outcomes much faster than destructive leader behaviors lower on hostility, like exploitative leadership. With exploitative leadership, negative effects, such as negative emotional reactions and turnover intentions, may only unfold over time.

Since our study focused on the individual follower perspective, future research needs to provide an understanding of how different destructive leader behaviors impact teams. We see different avenues for this. Peus et al. (2012) posited that the negative perceptions an individual develops of a leader can spread to the team through social and emotional contagion processes and create a shared negative perception of the leader. Thus, an employee who witnesses or becomes aware of the leader treating a colleague in an exploitative or abusive manner can be influenced by this (see also Priesemuth et al., 2014). Schmid et al. (2017) have shown first evidence for team level perceptions of exploitative leadership, but how these perceptions spread differently for different destructive leader behaviors remains to be understood. Moreover, followers may mimic their leader's behaviors (e.g., Yaffe and Kark, 2011). Further research should investigate how the different destructive behaviors may be mimicked and how follower mimicking abusive versus exploitative behaviors may impact teamwork. Related to this, future research should further investigate the role of followership in the destructive leader dynamic (Howell and Shamir, 2005). Followers may show these different destructive behaviors toward their leader; thus different destructive upward leadership behaviors and their outcomes need to be understood.

Moreover, when it comes to better understanding antecedents, leader identity and self-concept have received much attention in the leadership literature recently (e.g., Kark and Shamir, 2013; Mainemelis et al., 2015). It describes three levels of the self—the intrapersonal, the interpersonal, and the collective—and may be an important theory to understand why different destructive leaders behave in the way they do. We can imagine an exploitative leader focusing mainly on the intrapersonal aspect of self, whereas an abusive leader may rather focus on interpersonal aspects of their self. An organization-directed destructive leader would rather focus on the collective aspect.

In conclusion, a more tailored approach to destructive leadership, whether in research or practice, seems necessary, since all destructive leaders are destructive in their own way.

## Author contributions

All authors contributed extensively to the work presented in this paper. All authors designed the study jointly. ES and AP collected the data, AP conducted the majority of the data analysis. ES prepared the first draft of the manuscript, ES, AP, and CP wrote sections of the manuscript. ES, AP, and CP contributed to manuscript revisions, read and approved the submitted version.

### Conflict of interest statement

The authors declare that the research was conducted in the absence of any commercial or financial relationships that could be construed as a potential conflict of interest.

## Appendix

Scenarios used in Study 1

**Scenario: Follower-Directed Destructive Leadership***

Please imagine…

*You are working on a high priority project under a lot of time pressure. You have put a lot of effort into it and have achieved some very good results. Your boss, however, does not give you any credit for the hard work that was required or acknowledge the successful milestone you just completed, but rather keeps reminding you of the things that went wrong in the project. He makes fun of you in front of the whole project team and calls you a failure in public. When you make suggestions on how to go about the next project steps, he tells you that your ideas are stupid and even asks you not to interact with the other team members, since you are incompetent. He has broken numerous promises he made to you and it is not uncommon for him to lose his temper and shout*.

**Scenario: Self-Interested Destructive Leadership***

Please imagine…

*Your boss assigned you to a high priority project under a lot of time pressure. You have put a lot of effort into it, and were asked by your boss to work weekends and sacrifice training and professional development activities to reach the deadline. Now the project is completed and you are very proud of the outcome. Your boss, charming as ever, seizes the task of presenting the results to the customers, who are very impressed and invite him to present it at a prestigious convention. Your boss happily tells you that he has been invited on that all expenses paid trip. He gets all the fame for the successful project and, upon his return, even the desired promotion for advancing the company's reputation. You, however, do not get any credit for your work in the project*.

**Scenario: Organization-Directed Destructive Leadership***

Please imagine…

*You are working on a high priority project under a lot of time pressure. You have put a lot of effort into it, and have achieved some very good results*.

*During the course of the project, you realize that your boss is harming the organization. He regularly violates company policy. For example, he asks a colleague of yours, who is also working on the project, to take over private tasks for him, thus delaying her work on the project. Whilst reviewing project documentation, you realize that your boss has forged project results. Moreover, during informal talks with external business partners, he sometimes talks negatively about your organization. You heard him say what a lousy company this is. At a company dinner, you even saw him accept a corrupting gift. Recently, you also suspect him of sometimes coming to work under the influence of alcohol*.

**Please note: The original scenario was in German. This is a translation*.
